# ALA- and ALA-hexylester-induced protoporphyrin IX fluorescence and distribution in multicell tumour spheroids

**DOI:** 10.1054/bjoc.2001.1977

**Published:** 2001-09

**Authors:** C E Bigelow, S Mitra, R Knuechel, T H Foster

**Affiliations:** Institute of Optics; Departments of Biochemistry and Biophysics and; Radiology, University of Rochester, 601 Elmwood Avenue Box 648, Rochester, NY, 14642, USA; Institute of Pathology, University of Regensburg, Regensburg, D-93042, Germany

**Keywords:** aminolevulinic acid, aminolevulinic acid hexylester, protoporphyrin IX, multicell tumour spheroids, proliferation status

## Abstract

Synthesis of protoporphyrin IX (PpIX) in intact murine mammary cancer cell spheroids is reported from optical sections obtained using a laser scanning confocal fluorescence microscope. EMT6 spheroids 275–350 *μ* m in diameter were incubated in 0.1–15 mM aminolevulinic acid (ALA) or 0.001–2 mM ALA-hexylester (h-ALA) to test the ability of both pro-drugs to diffuse into the spheroids and induce PpIX production. Spheroids incubated with ALA show significant fluorescence nonuniformity for all concentrations, with the outermost cells exhibiting greater porphyrin fluorescence. Comparable levels of fluorescence throughout the optical section are achieved with approximately 100-fold lower h-ALA concentrations, indicating that the interior cells maintain esterase activity and porphyrin synthesis and that h-ALA diffuses efficiently to the spheroid interior. Fluorescence gradients are less pronounced with h-ALA incubation, in part because of apparent saturation of esterase activity in the spheroid perimeter. Proliferating (Ki67 positive) and quiescent cell populations exhibit remarkably different h-ALA concentration dependencies. The incubation concentration resulting in maximum fluorescence with ALA is 10 mM, while the optimal concentration for h-ALA is 200-fold lower at 0.05 mM. Exceeding these optimal concentrations for both pro-drugs leads to an overall loss of fluorescence. © 2001 Cancer Research Campaign http://www.bjcancer.com


bjoc 01-1977 727-734 20/8/01 3:44 pm Page 727

*British Journal of Cancer* (2001) **85**(5), 727–734
© 2001 Cancer Research Campaign
doi: 10.1054/ bjoc.2001.1977, available online at http://www.idealibrary.com on IDEAL

http://www.bjcancer.com

### ALA- and ALA-hexylester-induced protoporphyrin IX fluorescence and distribution in multicell tumour spheroids

**CE Bigelow[1], S Mitra[2], R Knuechel[4] and TH Foster[1,3]**

[1]Institute of Optics, Departments of [2]Biochemistry and Biophysics and [3]Radiology, University of Rochester, 601 Elmwood Avenue, Box 648, Rochester, NY 14642, USA; [4]Institute of Pathology, University of Regensburg, D-93042 Regensburg, Germany

**Summary** Synthesis of protoporphyrin IX (PpIX) in intact murine mammary cancer cell spheroids is reported from optical sections obtained using a laser scanning confocal fluorescence microscope. EMT6 spheroids 275–350 $\mu$m in diameter were incubated in 0.1–15 mM aminolevulinic acid (ALA) or 0.001–2 mM ALA-hexylester (h-ALA) to test the ability of both pro-drugs to diffuse into the spheroids and induce PpIX production. Spheroids incubated with ALA show significant fluorescence nonuniformity for all concentrations, with the outermost cells exhibiting greater porphyrin fluorescence. Comparable levels of fluorescence throughout the optical section are achieved with approximately 100-fold lower h-ALA concentrations, indicating that the interior cells maintain esterase activity and porphyrin synthesis and that h-ALA diffuses efficiently to the spheroid interior. Fluorescence gradients are less pronounced with h-ALA incubation, in part because of apparent saturation of esterase activity in the spheroid perimeter. Proliferating (Ki67 positive) and quiescent cell populations exhibit remarkably different h-ALA concentration dependencies. The incubation concentration resulting in maximum fluorescence with ALA is 10 mM, while the optimal concentration for h-ALA is 200-fold lower at 0.05 mM. Exceeding these optimal concentrations for both pro-drugs leads to an overall loss of fluorescence. © 2001 Cancer Research Campaign http://www.bjcancer.com

**Keywords**: aminolevulinic acid, aminolevulinic acid hexylester, protoporphyrin IX, multicell tumour spheroids, proliferation status

The pro-drug aminolevulinic acid (ALA) is the basis for a unique form of photosensitization that offers an alternative to strategies employing exogenous, preformed photosensitizers. ALA-photodynamic therapy (PDT) and fluorescence diagnostics rely on an accumulation of protoporphyrin IX (PpIX) in response to excess ALA overriding the rate-limiting step in heme synthesis. PpIX is an effective endogenous photosensitizer that can be used to detect and destroy a variety of lesions (Peng et al, 1997).

ALA efficiently induces PpIX production upon cellular uptake, but its hydrophilicity gives rise to poor transport across cell membranes. One approach to increase the effectiveness of ALA has been to develop various ALA esters with higher lipophilicity (Kloek and Beijersbergen van Henegouwen, 1996). Indeed it has been shown in excised tissue sections that aminolevulinic acid hexylester (h-ALA) increases tissue penetration depth and peak fluorescence relative to ALA (Marti et al, 1999). ALA esters also exhibit greater spatial confinement than ALA when topically applied to normal mouse skin (Peng et al, 1996). Further, the use of ALA esters reduces the ALA dose required for a given level of PpIX production (Uehlinger et al, 2000; Lange et al, 1999; Gaullier et al, 1997). However, PpIX production induced by ALA esters is complicated by the fact that the attached esters must be cleaved via cellular esterase activity before the ALA is available to the heme pathway (Kloek et al, 1998). This step can lead to differences in PpIX production in tissue exposed to ALA and h-ALA.

*Received 18 January 2001*
*Received 14 May 2001*
*Accepted 16 May 2001*

*Correspondence to:* TH Foster (thfoster@optics.rochester.edu)

PpIX synthesis in cells is affected by physiological conditions, cell type and proliferation status. Wyld et al (1997) observed a strong dependence in PpIX production with cell proliferation rate in endothelial cells, with proliferating cells accumulating more of the fluorophore than those that are quiescent. The oxygen tension and pH of the cellular environment were shown to affect the production of PpIX in exponentially growing bladder cancer cells (Wyld et al, 1998). Georgakoudi et al (1999) reported the effect of the combination of hypoxia, cell density and proliferation rate on PpIX production in EMT6 cells in monolayer cell culture. They demonstrated that PpIX is synthesized less effectively under hypoxia than in normoxic conditions, with the magnitude of the effect of hypoxia being strongly dependent on cell density and proliferation status. The complexity of the relationships among various factors influencing PpIX accumulation is reflected in a recent report of Krieg et al (2000), which showed that changes in PpIX content observed when normal urothelial, fibroblast and 2 human bladder cancer cell lines were cultured in subconfluent vs postconfluent conditions could be interpreted on the basis of intracellular iron content and ferrochelatase activity. In a well-differentiated tumour cell line, ferrochelatase activity increased significantly in postconfluent culture conditions, while in a poorly differentiated line, the ferrochelatase activity decreased when cell culture conditions were changed from subconfluent (exponential) to postconfluent (plateau).

In this study we report the ability of ALA and h-ALA to distribute and induce PpIX production in intact EMT6 multicell tumour spheroids. Spheroids are useful as an in vitro model of tumours in vivo because they possess three-dimensional spatial nonuniformities in oxygen concentration, proliferation status, metabolic activity, and nutrient availability. These inhomogeneities based on cells' location in the spheroid mimic the gradients established in solid tumours. PpIX production and distribution are quantified by fluorescence images acquired at a depth of 80–90 $\mu$m into the intact spheroids with a confocal fluorescence microscope.

### MATERIALS AND METHODS

### Chemicals

Aminolevulinic acid hydrochloride was purchased from Sigma (St Louis, MO, USA). h-ALA was kindly provided by Norbert Lange and Hubert van den Bergh at the Laboratory of Environmental Engineering, Swiss Federal Institute of Technology, Lausanne, Switzerland.

### Spheroid culture and pro-drug incubation

Our methods for spheroid culture are described more fully elsewhere (Foster et al, 1993) and are outlined briefly here. Approximately $10^6$ EMT6 cells are plated onto a 100 mm uncoated petri dish containing Eagle's basal medium (Gibco-BRL, Grand Island, NY, USA) and 10% fetal bovine serum (Atlanta Biologicals, Norcross, GA, USA). After incubation for 3 d at 37˚C in a humidified environment with a 5% $CO_2$–95% air atmosphere, the resulting aggregates are placed in a 500 ml spinner flask (Bellco Glass, Inc., Vineland, NJ, USA) containing 350 ml of the medium described earlier. From 3–5 d later, spheroids reach the 275–350 $\mu$m diameter range used in our experiments. Appropriately sized spheroids are removed from the flask and placed in uncoated petri dishes containing 20 ml of serum-free media with 0.1–15 mM ALA or 0.001–2 mM h-ALA. Sufficient buffering is available to maintain physiological pH. Spheroids are incubated with the pro-drugs for 3 h prior to imaging.

### Imaging of porphyrin fluorescence in intact spheroids

Detection and localization of PpIX fluorescence is performed by imaging thin optical sections of intact spheroids with an inverted laser scanning confocal fluorescence microscope of our own design and construction. Spheroids incubated with the pro-drugs are removed from the incubation medium and placed in a cover slip dish containing 1 ml of Hanks' Balanced Salt Solution (Gibco-BRL). PpIX fluorescence is excited with 514 nm light from an argon-ion laser. Fluorescence collected for imaging is discriminated from excitation light via reflection from a 585 nm short pass dichronic mirror (585 dcsp, Chroma Technology Corp., Brattleboro, VT, USA) and transmission through a long pass filter (KV550, Schott, Duryea, PA, USA) and is detected with a photomultiplier tube (HC 120-07 photosensor module, Hamamatsu, Bridgewater, NJ, USA). Output from the photomultiplier is filtered, amplified and digitized at 12 bits.

Images are acquired using a 500 $\mu$m × 500 $\mu$m field of view distributed over a 600 × 600 array of pixels resulting in an in-plane resolution of 0.83 $\mu$m/pixel. A 100 $\mu$m diameter pinhole rejects out-of-focus light, and a 10 ×, 0.5 NA air immersion microscope objective is used for excitation beam focussing and fluorescence collection. This combination of objective and pinhole gives an axial resolution of approximately 9 $\mu$m as determined by fluorescence edge response measurements. Location of the focus in the axial direction is accomplished using an axial focus controller with 100 nm resolution (MAC 2000, Ludl Electronic Products, Hawthorne, NY, USA).

Quantitative image reproducibility is ensured by daily calibrations. Images are corrected for the nonuniform response of the system across the field of view using data acquired from uniformly fluorescent fluid solutions. The peak fluorescence is checked to ensure that the signal retrieved from the reference sample does not deviate from one day to the next, and we verify that the edge response is consistent.

We analyse fluorescence images obtained at 80–90 $\mu$m from the bottom surface of the spheroids. The uncertainty in the exact location of the focus derives from imprecise knowledge of the refractive index of the spheroids, since the location of the optical section scales as the ratio of the sample index to the objective immersion index (Visser et al, 1992). This depth range is chosen because it is sufficiently deep to probe spatial fluorescence distribution nonuniformity while allowing for high-quality images. All spheroids are imaged in precisely the same manner, and the results are highly reproducible. Comparisons of confocal fluorescence images with images of thin frozen sections taken from similarly treated spheroids indicate that, at these depths and under our experimental conditions, attenuation due to absorption and scattering does not significantly distort the measured fluorescence distribution.

The porphyrin fluorescence distribution is quantified in the optical slices by partitioning each image into radial bins and reporting an average fluorescence value for each bin. Radial bins are delineated by superimposing concentric 12.5 $\mu$m-thick annular rings about the centre of the spheroid onto the image. As a gauge of total PpIX production, we also compute the average fluorescence generated throughout the entire optical section. In this case, the fluorescence values of all pixels within the image are summed and divided by the area of the spheroid slice.

### Statistical analysis of fluorescence distributions in confocal images

Pair-wise, two-tailed *t*-tests were performed to evaluate differences in fluorescence induced by different concentrations of ALA and h-ALA in each of the radial bins. *P*-values of 0.05 or less were considered significant.

### Microelectrode measurements of oxygen concentrations

To identify possible oxygen-dependent effects on prophyrin synthesis, oxygen concentration measurements are made using 12 $\mu$m tip diameter Clark-style microelectrodes (Diamond General Development Corp., Ann Arbor, MI, USA) on spheroids incubated in drug-free solution, 5 mM ALA, 0.05 mM h-ALA, and 1 mM h-ALA. These pro-drug concentrations are chosen because, from the standpoint of porphyrin accumulation, they represent nearly optimal incubation concentrations and a concentration above the optimum value. Details of the measurement and analysis procedures are described fully elsewhere (Nichols and Foster, 1994).

### PpIX redistribution

PpIX redistribution during incubation could influence fluorescence distributions observed in the images. We tested for redistrib-

 *© 2001 Cancer Research Campaign*

ution with spheroids incubated in 1 mM h-ALA, as these possess a substantial fluorescence nonuniformity that would make evident PpIX relocation. Spheroids were first incubated for 3 h using the procedures outlined previously. Upon removal from the incubator, several spheroids were selected and imaged immediately. The remaining spheroids were placed in drug-free solution in a refrigerator at 8˚C to minimize metabolic activity and further porphyrin synthesis. PpIX fluorescence in the refrigerated spheroids was imaged at 1 h intervals for 3 h and at longer intervals up to 24 h after the onset of cold incubation. Changes in the radial distribution of fluorescence observed at these later timepoints could therefore be attributed predominantly to redistribution of PpIX synthesized during the initial 3-hour incubation.

### Immunohistochemical staining of spheroid cell proliferation status

The proliferation status of cells throughout the spheroids is determined by staining physically sectioned spheroids with an antibody against Ki67. Expression of Ki67 is associated with proliferation (Scholzen and Gerdes, 2000). Serial sections of spheroids (5 $\mu$m-thick) were cut and placed on coated glass slides (SuperFrost Plus, Menzel Gläser, Stuttgart, Germany). After microwave pretreatment for 15 min in 10 mM citrate buffer, sections were stained with an antibody against Ki67 (rat-anti-mouse Ki67, clone TEC-3, Dianova, Hamburg, Germany) using an indirect immunoperoxidase technique. Standard blocking and washing procedures were carried out prior to or after the antibody incubations, respectively. The first antibody was used at a concentration of 1:10 in PBS for 1 h at room temperature. The second biotinylated anti-rat antibody (Jackson Immuno Research Inc., West Grove, PA, USA) was applied at a concentration of 1:200 for 30 min, followed by incubation with a biotinylated peroxidase complex (DAKO, Hamburg, Germany) for 30 min at room temperature. Substrate incubation (Diaminobenzidine, DAKO) was carried out for 5 min at room temperature, followed by further counterstaining with haematoxylin for 5 min, dehydration of sections, and mounting. Photomicrographs of sections were acquired on an Aristoplan microscope (Zeiss, Jena, Germany) using a 640 ASA film (Scotch Chrome, Segrate, Italy).

### RESULTS

Figure 1 shows representative confocal fluorescence images of PpIX fluorescence in EMT6 spheroids. Images shown in Figure 1 A–C were incubated in varying concentrations of ALA; those shown in Figure 1 D–F were incubated in a range of h-ALA concentrations. The radial distributions of PpIX fluorescence in spheroids exposed to various concentrations of ALA are shown in Figure 2A and B. Spheroids incubated in 0.1 mM ALA yield very low fluorescence signals, although the magnitude of the signal produced is significantly elevated above autofluorescence throughout the section (Figure 2A). As the ALA concentration is increased, there is a significant increase in fluorescence at all radii. The outer rim porphyrin fluorescence increases more quickly than does that of the inner regions, which leads to marked non-uniformity for intermediate concentrations. Spheroids incubated in 10 mM ALA exhibit a maximum in distribution non-uniformity,

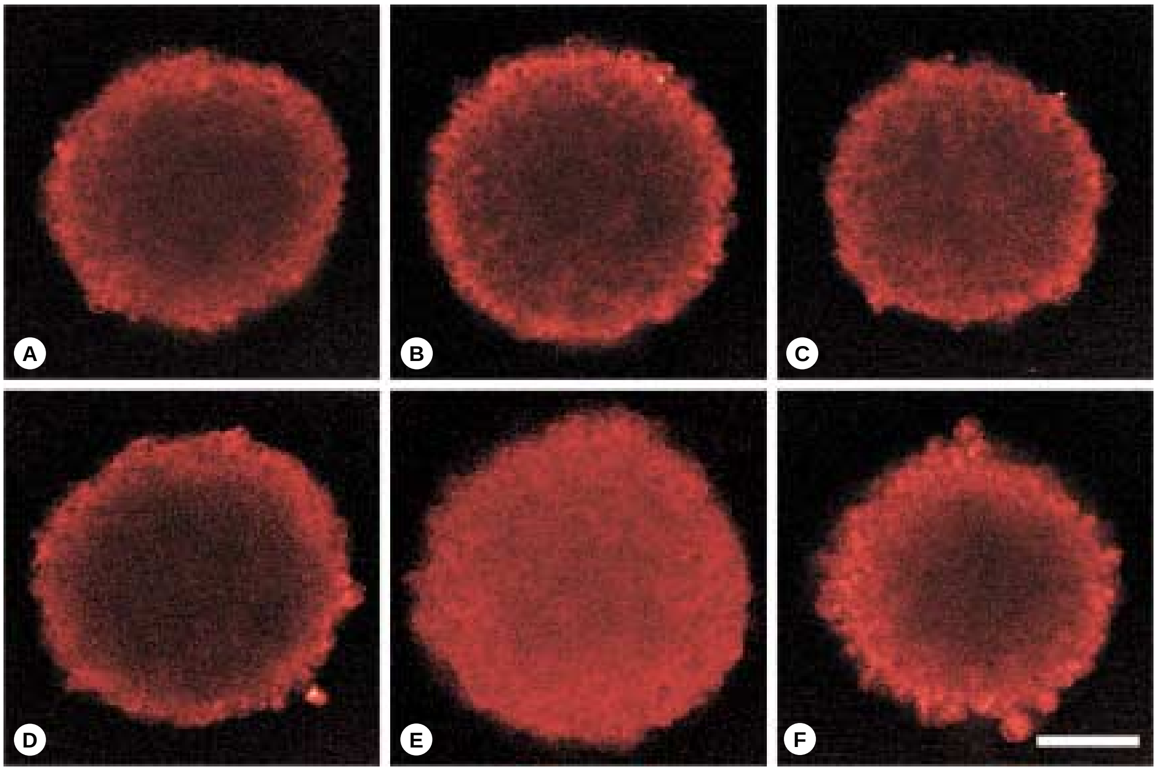

**Figure 1** Confocal fluorescence microscope images acquired at a depth of 80–90 $\mu$m from spheroids incubated in ALA at concentrations of (**A**) 0.5 mM, (**B**) 2 mM and (**C**) 10 mM or in h-ALA at concentrations of (**D**) 0.005 mM, (**E**) 0.05 mM and (**F**) 1 mM. The optical section thickness is approximately 9 $\mu$m and the in plane resolution is 0.8 $\mu$m. The bar in (**F**) corresponds to 100 $\mu$m

*© 2001 Cancer Research Campaign* 

bjoc 01-1977 727-734  20/8/01  3:44 pm  Page 730

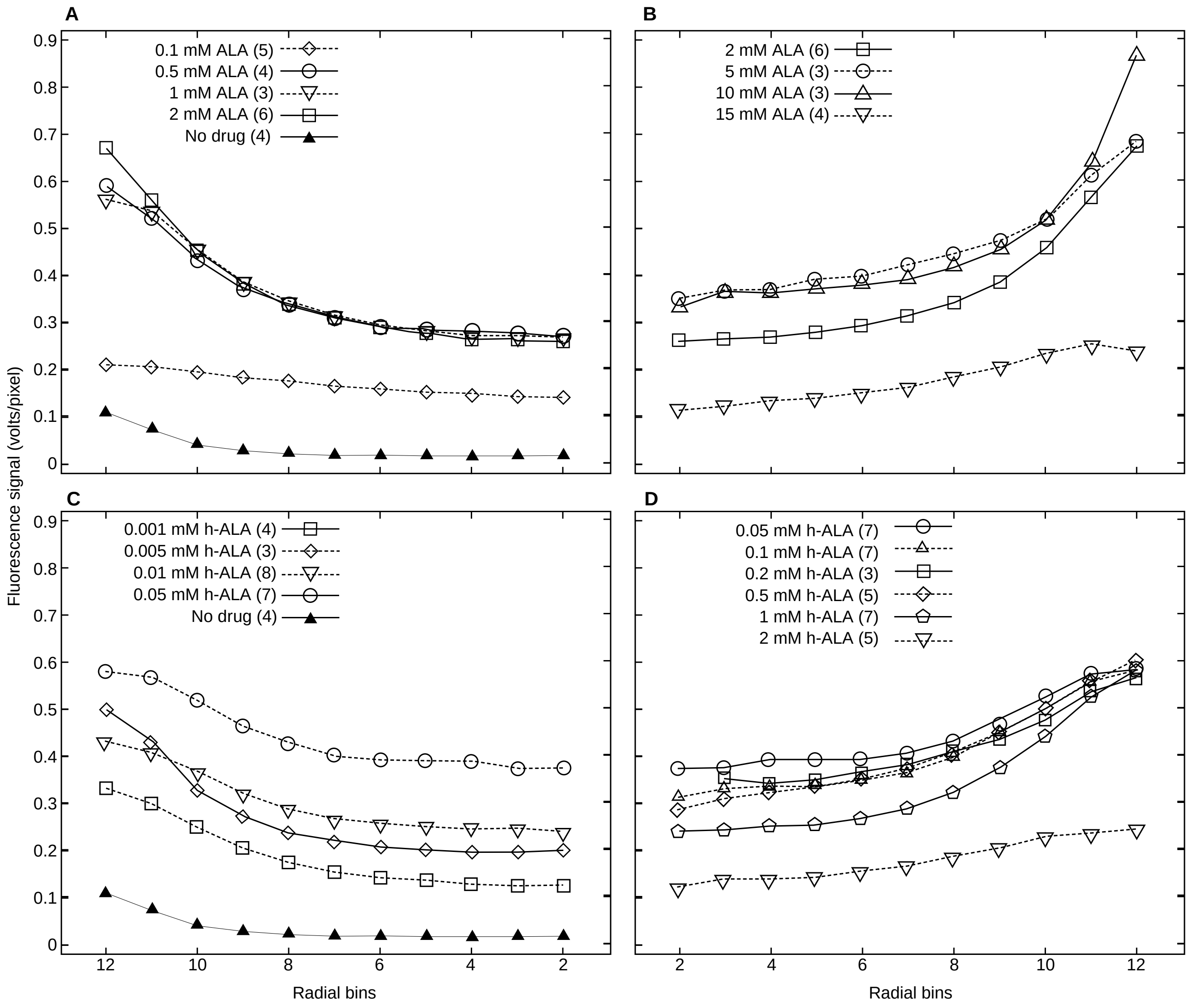

**Figure 2** Radial fluorescence distributions in confocal fluorescence images for spheroids incubated for 3 h with 0.1–15 mM ALA (**A, B**) and 0.001 mM–2 mM h-ALA (**C, D**). Error bars are not shown for clarity. The most central region of the spheroids corresponds to radial bin 2, and the outer rim corresponds to radial bin 12. The number of spheroids imaged at each incubation concentration is displayed in parentheses. (**A**) Control spheroids and 0.1–2 mM ALA; (**B**) 2–15 mM ALA. The spheroid outer rim produces more fluorescence than the central region at all ALA concentrations. Fluorescence signal increases at a higher rate in the perimeter as the concentration is increased so that at 10 mM the radial PpIX fluorescence gradient is most pronounced. Concentrations greater than 10 mM result in reduced fluorescence at all radii. (**C**) Control spheroids and 0.001–0.05 mM h-ALA; (**D**) 0.05–2 mM h-ALA. Fluorescence amplitudes at all radii increase with concentrations up to 0.05 mM, but from 0.05–1 mM the outer rim fluorescence remains constant at 0.58 V/pixel. Concentrations above 0.05 mM introduce a reduction in fluorescence in the interior regions and eventually lead to a fluorescence decrease throughout the optical section with the outermost rim exhibiting nearly 3-fold more fluorescence than in the centre (Figure 2B). Concentrations above 10 mM lead to a dramatic reduction in fluorescence throughout the spheroid.

The radial fluorescence distributions for spheroids incubated with h-ALA are shown in Figure 2C and D. The fluorescence gradient and the maximum fluorescence are highly dependent on the h-ALA incubation concentration. For the lowest concentrations (0.001 mM), the radial distribution of PpIX in the spheroids is non-uniform with approximately 3-fold greater fluorescence in the outermost radial bin as compared to the central bins (Figure 2C). The fluorescence signal in the central regions is comparable to that observed in response to the lowest ALA concentration of 0.1 mM, while in the periphery the fluorescence induced by 0.001 mM h-ALA exceeds that induced by 0.1 mM ALA by 50%. As the h-ALA concentration is increased, the fluorescence in all regions of the spheroids responds by increasing as well. At 0.05 mM h-ALA, the fluorescence reaches its most uniform radial distribution, with only a 25% increase from the spheroid centre to the outermost rim. This is the most uniform of any of the incubation conditions tested that produces significant signal, including those with ALA. This result indicates that all regions of these spheroids at the 80–90 $\mu$m depth tested are able to maintain esterase activity and porphyrin synthesis under the given conditions. It also indicates that the h-ALA is penetrating efficiently to the interior regions and inducing PpIX production. As the h-ALA concentration increases from 0.05 mM to 1 mM, an interesting progression occurs. The spheroid fluorescence in the most peripheral radial bin becomes fixed at a value of about 0.58 V/pixel, while the interior regions show a decrease in fluorescence intensity (Figure 2D). The modest reductions in fluorescence observed in the inner radial bins

 © 2001 Cancer Research Campaign at 0.1 and 0.2 mM h-ALA are not statistically significant. In response to 0.5 mM h-ALA, the fluorescence measured in the most central radial bin (bin 2) is significantly lower than that measured in the same bin at 0.05 mM ($P < 0.05$). When the concentration is increased to 1 mM h-ALA, the fluorescence throughout the image is significantly reduced, except for the most peripheral radial bin (bin 12). The result is that the non-uniformity in porphyrin fluorescence is reintroduced, such that at 1 mM the gradient in fluorescence distribution is once again quite substantial. Increasing the h-ALA concentration from 1 mM to 2 mM causes the overall fluorescence to drop in a manner similar to that shown with the highest ALA concentrations (10–15 mM). The progression from an initially non-uniform PpIX distribution to one that is quite uniform and back again is illustrated in Figure 1 (D–F), where images are presented of spheroids incubated with 0.005 mM, 0.05 mM and 1 mM h-ALA.

Figure 3 displays the average fluorescence in the optical sections for the entire range of incubation concentrations for ALA and h-ALA. Both plots share a qualitatively similar increase and subsequent decrease in fluorescence with pro-drug concentration that agrees well with results obtained from other cell lines in monolayer cell culture (Uehlinger et al, 2000). The maximum value of the total PpIX fluorescence signal for h-ALA is nearly equal to that observed for ALA. However, the peak fluorescence is achieved for h-ALA at 100–200-fold lower incubation concentrations than for ALA. It is apparent from Figures 2 and 3 that the range of h-ALA concentrations that yields the maximum average fluorescence also produces the most uniform distributions. In contrast, the peak fluorescence for ALA occurs at 10 mM, which produces the most pronounced radial fluorescence gradient.

Oxygen concentration profiles from spheroids incubated in 0.05 mM and 1.0 mM h-ALA are shown in Figure 4A and B, respectively. The medium in which the spheroids are immersed during the electrode measurements has an air-saturated oxygen concentration of 240 $\mu$M at distances greater than 300 $\mu$m from the edge of the spheroids, which corresponds to a radial distance of approximately 460 $\mu$m in the plots of Figure 4. Closer to the spheroid, oxygen levels are decreased due to metabolic consumption. The oxygen gradient steepens as the electrode penetrates the spheroid at a radius of roughly 150–160 $\mu$m. For both of these h-ALA incubation concentrations, the oxygen concentrations everywhere in the spheroids remain well above levels at which PpIX synthesis would be influenced (Wyld et al, 1998). The average rates of metabolic oxygen consumption in the spheroids determined from analysis of the electrode measurements indicate that incubation with either pro-drug in this concentration range introduces only a modest perturbation in oxygen consumption (data not shown).

The redistribution experiments summarized in Figure 5 show that the PpIX fluorescence does not relocalize significantly in spheroids incubated in 1 mM h-ALA. The highly non-uniform fluorescence of the 1 mM h-ALA-incubated spheroids remains approximately constant for as long as 24 h after the initial 3-hour incubation. The fluorescence in the outermost bin remains constant within experimental error for the duration of these experiments. A maximum increase of 28% in the central regions occurs at the 6-hour time point, with later times yielding slightly lower fluorescence signals. The absolute increase and subsequent decrease in fluorescence with time is roughly constant for all but the most peripheral radial bins, consistent with some continued haem synthesis during cold incubation. No net redistribution of PpIX is evident in these data, however.

Figure 6 shows a photomicrograph of a 5 $\mu$m-thick representative spheroid central cross-section stained for tumour cell proliferation status using an antibody directed against Ki67. The proliferating fraction, which displays brown nuclei as a result of the immunoperoxidase reaction, is confined to the periphery. While the number of proliferating cells varies among spheroids, their predominance in the outer rim is consistent. The blue colour in the figure derives from counterstaining with hematoxylin. Cells within 70 $\mu$m of the spheroid centre possess smaller, darker nuclei, indicative of necrosis in the spheroid centre. The approximate depth of the optical section is indicated by the pair of horizontal lines. The 80–90 $\mu$m-deep confocal image thus samples two distinct cell populations: the proliferating cells in the periphery and the quiescent but viable cells in the interior. The images do not, however, probe the central necrotic region.

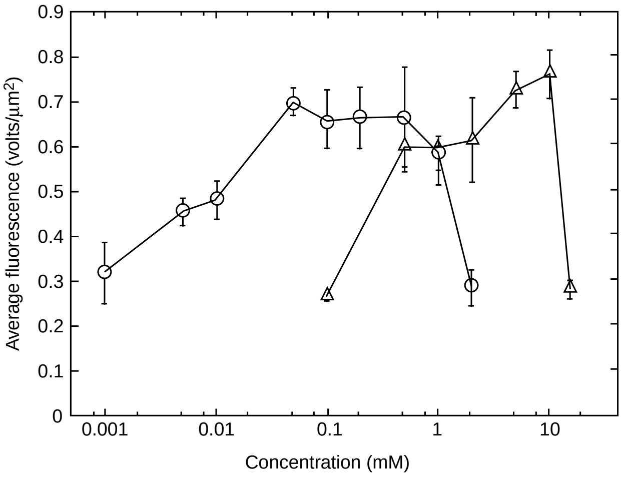

**Figure 3** Average fluorescence (V/$\mu m^2$) for 80–90 $\mu$m-deep optical sections for spheroids treated with ALA (△) and h-ALA (○). ALA-incubated spheroids exhibit maximum PpIX fluorescence at 10 mM, while h-ALA incubation yields maximal fluorescence at 0.05 mM. Both pro-drugs induce nearly the same maximum fluorescence signal

### DISCUSSION

Responses of cells at various locations in the h-ALA-and ALA-incubated spheroids provide insight into the mechanisms that govern and limit ALA uptake and conversion to PpIX. For example, Figure 2C shows that for h-ALA concentrations up to 0.05 mM, porphyrin fluorescence throughout the optical section increases with increasing pro-drug concentration. It is therefore reasonable to conclude that ALA availability is the limiting factor in PpIX production at these low h-ALA concentrations. In the intermediate range of h-ALA concentrations from 0.05 mM to 1 mM (Figure 2D), the fluorescence in the most peripheral radial bin remains very nearly constant at a value of approximately 0.58 V/pixel, while the fluorescence accumulated in the central regions decreases significantly when the h-ALA concentration is increased from 0.05 to 1 mM. Cells at the same outermost radial location in spheroids incubated with ALA do not exhibit this same level of saturation in fluorescence. At an ALA concentration of 10 mM, the fluorescence in the cells at the rim reaches a value of 0.87 V/pixel, more than 30% greater than the maximum observed

*© 2001 Cancer Research Campaign*

bjoc 01-1977 727-734 20/8/01 3:44 pm Page 732

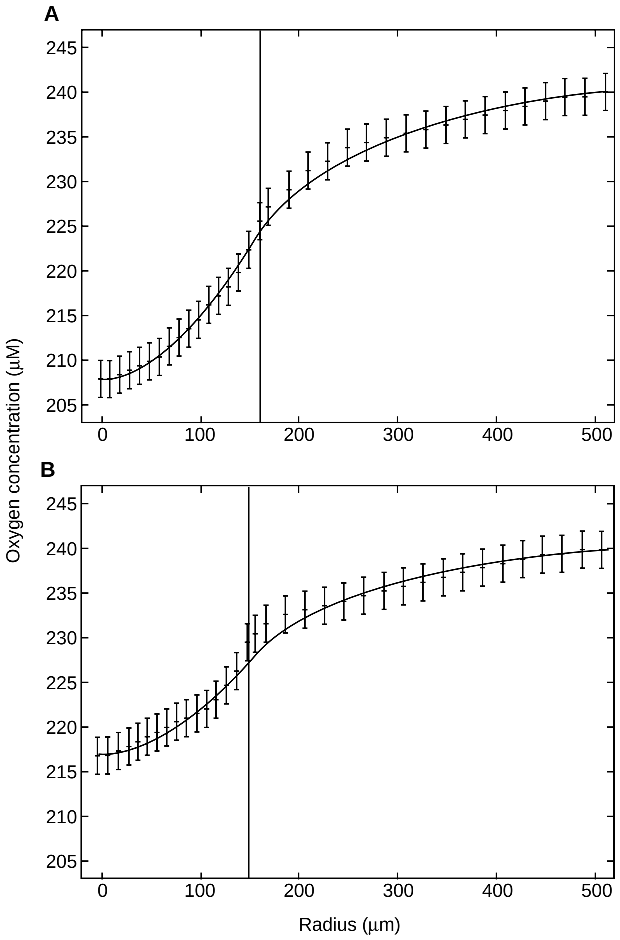

**Figure 4** Oxygen microelectrode measurements for (**A**) a spheroid incubated with 0.05 mM h-ALA and (**B**) with 1 mM h-ALA. Electrode data are displayed as points with the errors bars indicating electrode measurement uncertainty. The origin of the horizontal axis corresponds to the centre of the spheroid. The solid lines indicate a fit to the data using numerical solutions to a diffusion with consumption equation. The vertical lines at 160 (**A**) and 150 (**B**) $\mu$m indicate the surface of the spheroid

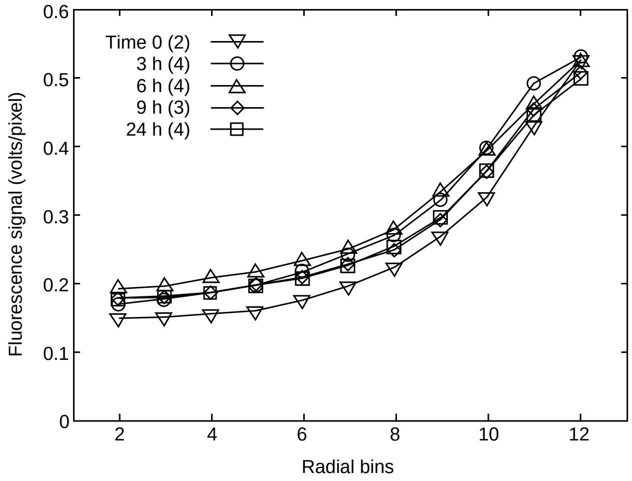

**Figure 5** Fluorescence redistribution data from spheroids incubated in 1 mM h-ALA for an initial 3 h period ($t$ = 0) and subsequently subjected to cold incubation. The fluorescence in the outermost rim remains constant within experimental error, while the interior increases by a maximum of 28% at the 6 h time point and then diminishes. The fluorescence distribution remains nearly constant up to 24 h from the onset of cold incubation in h-ALA-incubated spheroids. This difference in porphyrin accumulation in the proliferating fraction is consistent with the conclusion that, in the intermediate concentration range, it is the membrane esterase activity in the outermost cells of h-ALA-incubated EMT6 spheroids that limits PpIX production rather than saturation of the haem biosynthetic pathway. Saturation of esterase activity also seems to play a role in establishing the more uniform spatial distribution of porphyrin fluorescence observed in response to 0.05 mM h-ALA. Thus the improved uniformity in fluorescence accomplished with h-ALA as compared to ALA follows in part from a decrease in the maximum achievable fluorescence near the periphery of the spheroid.

As noted earlier, for h-ALA incubation concentrations between 0.05–1.0 mM, the fluorescence signal originating from quiescent cells in the interior of the optical section decreases in response to increased amounts of the pro-drug, while the fluorescence in the proliferating fraction near the rim remains constant (Figure 2D). This diminishing signal with increased concentration indicates that these non-proliferating cells are showing relatively greater toxicity to higher h-ALA concentrations, suggesting the possibility of some form of proliferation-status-dependent sensitivity to either

 *© 2001 Cancer Research Campaign*

bjoc 01-1977 727-734 20/8/01 3:44 pm Page 733

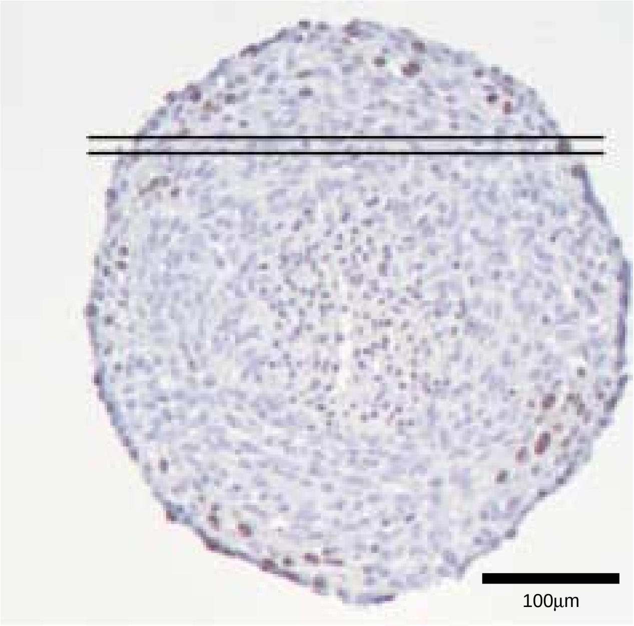

**Figure 6** A representative 5 $\mu$m-thick histologic section of an EMT6 spheroid stained to reveal proliferation status. Dark-brown staining in the figure results from the immunoperoxidase reaction and indicates the presence of Ki67 in proliferating cells. These cells are confined to the outermost portion of the viable rim of the spheroid. The region imaged by the confocal microscope is indicated by the pair of solid horizontal lines through the spheroid the ALA itself or to the alcohol resulting from the enzymatic cleavage of the hexylester. The reason for this difference in toxicity is unknown, but it underscores the point that the responses of heterogeneous populations of cells to a given concentration of h-ALA are likely to be significantly different. An overall reduction in fluorescence is observed throughout the spheroids beyond a certain concentration of both ALA and h-ALA, which is consistent with results reported by Uehlinger et al (2000) for several human cell lines in monolayer culture. This response to high concentrations of both pro-drugs is similar, therefore it must be caused by the ALA itself. Mechanisms of ALA dark toxicity have been reviewed by Peng et al (1997). In our experiments, the concentrations of ALA and h-ALA at which this overall reduction was evident were 15 and 2 mM, respectively. For the ALA case, 15 mM is just 50% higher than the 10 mM concentration that produced the maximum in PpIX fluorescence throughout the optical section (Figure 3). For h-ALA on the other hand, the 2 mM concentration is 40-fold higher than the 0.05 mM incubation concentration that produced maximum fluorescence. Thus, another advantage of the use of h-ALA seems to be a wider ‘window’ of concentrations at which nearly optimal fluorescence is achieved. This phenomenon is likely to be another consequence of the limit imposed by esterase activity on the rate at which ALA is delivered to the interior of the cells.

The fluorescence gradients reported here appear to result from a combination of factors including differences in PpIX production, a disparity in spheroid penetration between ALA and h-ALA, the rate of enzymatic cleavage of the ester, and different dark toxicities in proliferating vs quiescent cell populations. Experiments were performed to exclude possible effects of porphyrin redistribution and hypoxia. Our redistribution experiments, summarized in Figure 5, show no significant relocalization of PpIX, even on timescales much longer than the 3-hour time point evaluated in this study. Over a 24-hour period, fluorescence changes in optical sections acquired from spheroids subjected to cold incubation (after the initial 3-hour warm incubation) are modest. More importantly, the slight increase and subsequent decrease in fluorescence occurs in all but the most peripheral of the radial bins, consistent with some continued porphyrin synthesis and heme formation throughout the spheroid on this timescale. If significant redistribution were occurring, increased fluorescence in the inner locations would be accompanied by decreased fluorescence at the periphery. The fact that this is not observed means that redistribution of porphyrins, once formed, is not important in this system, even when the initial fluorescence distribution is highly non-uniform, as it was in this experiment. We also cannot attribute diminished interior spheroid fluorescence to regions of reduced oxygen concentrations, because direct measurement shows that the spheroid centres remain well above the hypoxic levels that would limit porphyrin synthesis.

In summary, PpIX synthesis occurs throughout EMT6 spheroids exposed to ALA and the derivative h-ALA. The magnitude of h-ALA-induced fluorescence is comparable to that induced by ALA at 100–200-fold higher concentrations. PpIX distributions resulting from h-ALA at optimal concentrations are more uniform than those observed with ALA at optimal concentrations. Membrane esterase activity is apparently involved in limiting PpIX synthesis in the spheroid periphery, which contributes to the enhanced uniformity. The rate of enzymatic cleavage also imparts a measure of protection against the onset of dark toxicity, creating a wider range of h-ALA concentrations that induce nearly optimal fluorescence. Nevertheless, caution must be used in the application of h-ALA. The efficiency with which it is taken up by cells requires the use of far lower concentrations than are required with ALA.

### ACKNOWLEDGEMENTS

The authors would like to thank Norbert Lange and Hubert van den Bergh of the Laboratory of Environmental Engineering at the Swiss Federal Institute of Technology for providing ALA hexylester for this study. We are also pleased to acknowledge Helmut Kutz for performing the Ki-67 staining of the EMT6 spheroid sections. This work was supported by National Institutes of Health grants CA68409 and CA36856.

### REFERENCES

Foster TH, Hartley DF, Nichols MG and Hilf R (1993) Fluence rate effects in photodynamic therapy of multicell tumor spheroids. *Cancer Res* **53**: 1249–1254

Gaullier JM, Berg K, Peng Q, Anholt H, Selbo PK, Ma L and Moan J (1997) Use of 5-aminolevulinic acid esters to improve photodynamic therapy on cells in culture. *Cancer Res* **57**: 1481–1486

Georgakoudi I, Keng PC and Foster TH (1999) Hypoxia significantly reduces aminolaevulinic acid-induced protoporphyrin IX synthesis in EMT6 cells. *Br J Cancer* **79**: 1372–1377

Kloek J, Akkermans W and Beijersbergen van Henegouwen GMJ (1998) Derivatives of 5-aminolevulinic acid for photodynamic therapy: enzymatic conversion into protoporphyrin. *Photochem Photobiol* **67**: 150–154

Kloek J and Beijersbergen van Henegouwen GMJ (1996) Prodrugs of 5-aminolevulinic acid for photodynamic therapy. *Photochem Photobiol* **64**: 994–1000

Krieg RC, Fickweiler S, Wolfbeis OS and Knuechel R (2000) Cell-type specific protoporphyrin IX metabolism in human bladder cancer *in vitro*. *Photochem*

© 2001 Cancer Research Campaign 

bjoc 01-1977 727-734 20/8/01 3:44 pm Page 734

*Photobiol* **72**: 226–233
Lange N, Jichlinski P, Zellweger M, Forrer M, Marti A, Guillou L, Kucera P, Wagnieres G and van den Bergh H (1999) Photodetection of early human bladder cancer based on the fluorescence of 5-aminolaevulinic acid hexylester-induced protoporphyrin IX: a pilot study. *Br J Cancer* **80**: 185–193
Marti A, Lange N, van den Bergh H, Sedmera D, Jichlinski P and Kucera P (1999) Optimisation of the formation and distribution of protoporphyrin IX in the urothelium: an in vitro approach. *J Urol* **162**: 546–552
Nichols MG and Foster TH (1994) Oxygen diffusion and reaction kinetics in the photodynamic therapy of multicell tumor spheroids. *Phys Med Biol* **39**: 2161–2181
Peng Q, Berg K, Moan J, Kongshaug M and Nesland JM (1997) 5-Aminolevulinic acid-based photodynamic therapy: principles and experimental research. *Photochem Photobiol* **65**: 235–251
Peng Q, Moan J, Warloe T, Iani V, Steen HB, Bjorseth A and Nesland JM (1996) Build-up of esterified aminolevulinic-acid-derivative-induced porphyrin fluorescence in normal mouse skin. *J Photochem Photobiol B* **34**: 95–96
Scholzen T and Gerdes J (2000) The Ki-67 protein: from the known and the unknown. *J Cell Physiol* **182**: 311–322
Uehlinger P, Zellweger M, Wagnieres G, Juillerat-Jeanneret L, van den Bergh H and Lange N (2000) 5-Aminolevulinic acid and its derivatives: physical chemical properties and protoporphyrin IX formation in cultured cells. *J Photochem Photobiol B* **54**: 72–80
Visser TD, Oud JL and Brakenhoff GJ (1992) Refractive index and axial distance measurements in 3-D microscopy. *Optik* **90**: 17–19
Wyld L, Burn JL, Reed MWR and Brown NJ (1997) Factors affecting aminolaevulinic acid-induced generation of protoporphyrin IX. *Br J Cancer* **76**: 705–712
Wyld L, Reed MWR and Brown NJ (1998) The influence of hypoxia and pH on aminolaevulinic acid-induced photodynamic therapy in bladder cancer cells in vitro. *Br J Cancer* **77**: 1621–1627

 *© 2001 Cancer Research Campaign*
